# Supplementary material for: The Processivity of Telomerase: Insights from Kinetic Simulations and Analyses
Source: Molecules. 2021 Dec 13;26(24):7532. doi: 10.3390/molecules26247532 (PMC8705835; doi:10.3390/molecules26247532)
Supplement: Supplementary file 1 [file molecules-26-07532-s001.zip › molecules-1418739-supplementary.pdf]

molecules MDPI

Supplementary material

The processivity of telomerase: insights from kinetic simulations and analyses

Clive R. Bagshaw[1,*], Jendrik Hentschel[1,2] and Michael D. Stone[1]

[1] Department of Chemistry and Biochemistry, University of California at Santa Cruz, Santa Cruz, CA 95064, USA
[2] Present address: Element Biosciences, 9880 Campus Point Drive, San Diego, CA 92121, USA
* Correspondence: CRB, cbagshaw@ucsc.edu; MDS, mds@ucsc.edu

I) Supplementary Figures

Figure S1 Analysis of primer extension assays

Figure S2 Analysis of NA-bands

II) Supplementary Tables

Table S1 Analysis of resolved NA-bands

Table S2 Analysis of pooled NA-bands

Table S3 Analysis of RA-bands only

III) Example software scripts

Figure S1

(a)

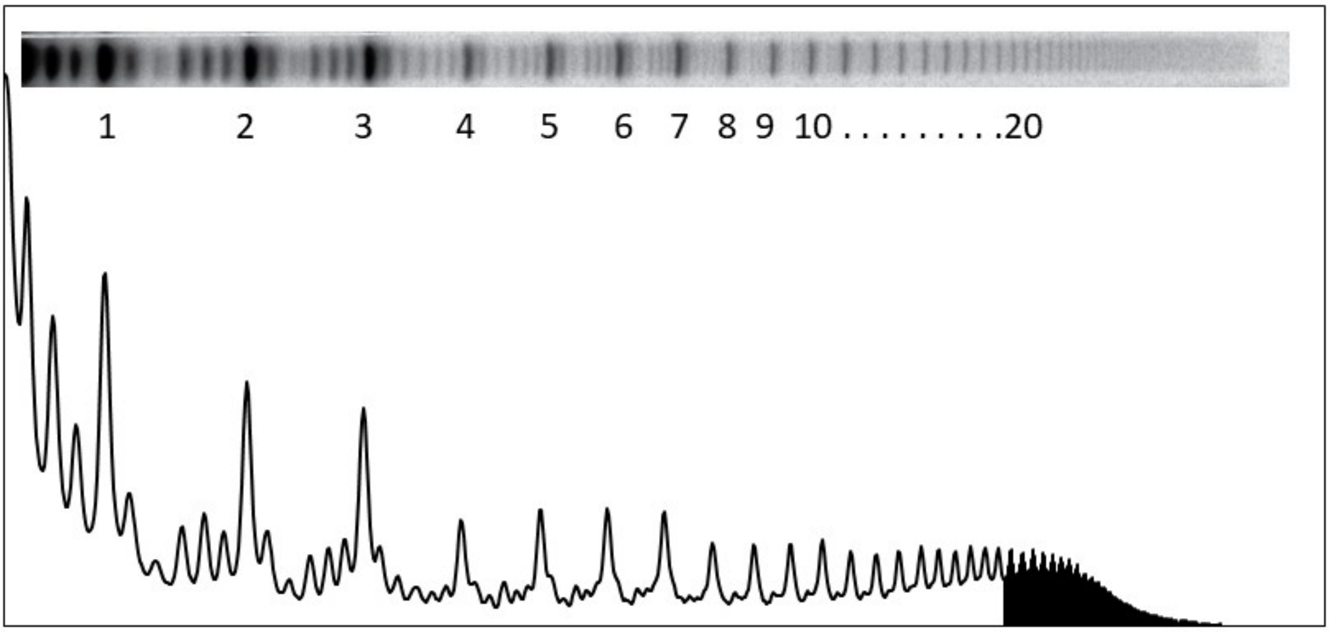

(b)

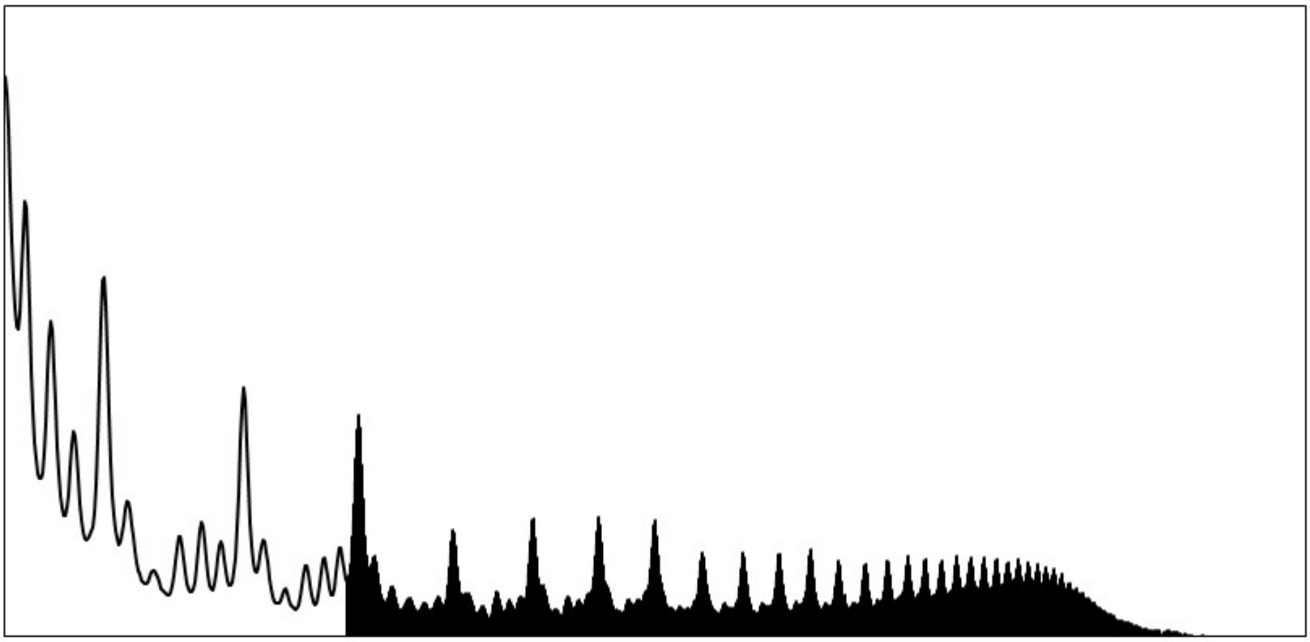

Figure S1. Example of a labeled primer extension assay monitored by gel electrophoresis to illustrate the challenge of analysing band intensities. (a) The strong bands, labelled 1 to 20 are the RA-bands characteristic of Repeat Addition Processivity (RAP). The intervening weaker NA-bands represent nucleotide addition processivity (NAP) and are only

resolved upto about the 5th RA-band. Between RA-bands 6 to 14 the NA-band intensity is evident, but could only be quantfied as pooled intensity. Beyond RA-band 14, NA-band intensity is not resolved from the surrounding RA-bands, while beyond RA-band 20, the RA-bands are poorly resolved. Nevertheless the total intensity beyond RA-band 20 respresents a significant fraction of the total (~10% in this example) which, if ignored, leads to significant systematic deviation in the estimated processivity in some normalization procedures (main text Figure 2c,d). Note also the strong intensity of the first 3 RA-bands, followed by two clusters of 4 indicative of G-quadruplex modulation {Jansson, 2019 #7946}. (b) Unbiased $R_{1/2}$ estimate based on cumulative intensity showing 50% of the band intensity is associated with products corresponding to the 3rd RA-band and beyond. The cumulative intensity and its 50% value was calculated in a spreadsheet of the digitized gel scan.

Figure S2

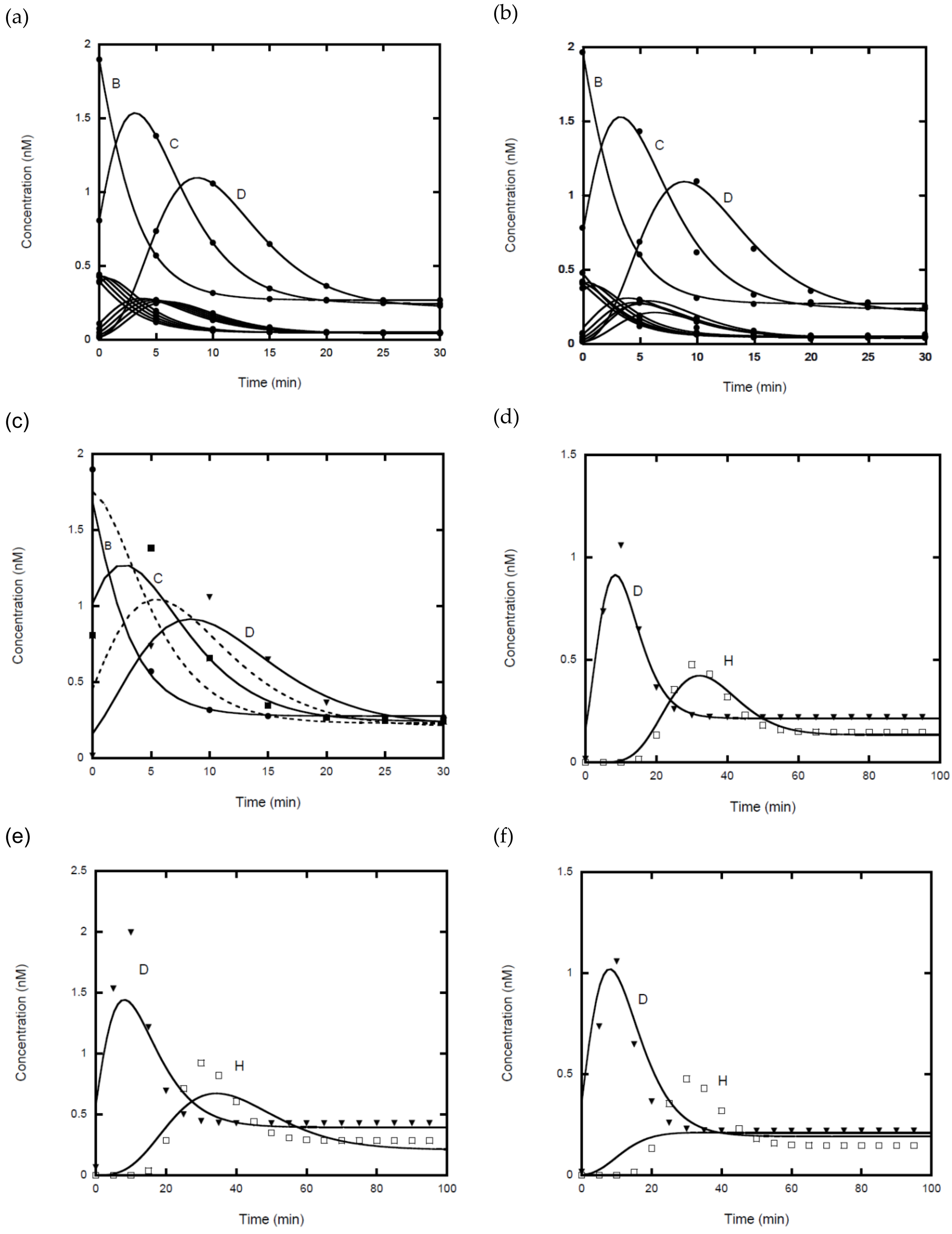

Figure S2. Analysis of simulated data to show the effect of different treatments of the NA-band intensities (a) DynaFit analysis (solid line) of simulated data (solid circles) for the model in Figure 6b showing that even with relatively sparse sampling (every 5 minutes), the input rate constants can be accurately fitted for the NA- and RA-transitions provided there is no noise in the data (Table S1). The NA-transitions preceding the first RA-transition are complete within 5 min and omitted from the plot. The first 3 RA-bands are indicated B, C and D (cf. Figure 7a). (b) Similar analysis but with data set in which ± 10% random noise was added to each concentration (see text) to test the effect on the estimated rate

constants. (c) Attempted fit to a simplified model in which the five NA-transitions between each RA-event are treated as a single step (shown as dashed lines) as may be appropriate for experimental data where the NA-bands are poorly resolved. The solid fitted lines to the RA-bands no longer pass through all the data points (circle = B, squares = C and triangle = D), although the extracted rate constants for the RA-events are fairly close to the input values (Table S2). (d) Similar analysis for the 3rd (solid triangle) and 7th (open squares) RA-intensities (bands D and H) to show deviations. Note that the weighting of the fit shifts from the end point values for the early RA-bands to the transient concentration values for later RA-bands. (e) An example fit to 3rd and 7th RA-bands where the NA-intensities are summed with the adjacent RA-intensities, as would occur experimentally when the NA-bands are no longer resolved from the RA-bands. The derived rate constants for all RA-transitions are summarized in Table S3. (f) Fit to 3rd and 7th RA-bands in which all NA-band intensities were ignored. The fit deviates widely for the 7th RA-band and accounts for the poor estimate of k_{d7} and k_{f7} in Table S3. Ignoring the NA-bands gives an increasing deviation with repeat number, including endpoint values, because mass is no longer conserved.

Table S1. DynaFit analysis of simulation of Figure S2a,b in which both RA- and NA-bands were analysed. RA-band data shown in bold. The microscopic processivity was calculated from $k_f/(k_f + k_d)$ at each step.

Repeat number	Rate constant	Input value (min^{-1})	DynaFit estimate (+ 0% noise)	DynaFit estimate (+/- 10% noise)	Microscopic Processivity (+/- 10% noise)
	NA- k_{f01}	1.86500	1.83439	1.19639	
	NA- k_{d02}	0.01865	0.01878	0.02324	
	NA- k_{f02}	1.86500	1.87623	2.33084	0.990
	NA- k_{d03}	0.01865	0.01876	0.02269	
	NA- k_{f03}	1.86500	1.87365	2.27286	0.990
**1**	**RA- k_{d1}**	**0.01865**	**0.01869**	**0.01908**	
	**RA- k_{f1}**	**0.34780**	**0.34810**	**0.35444**	**0.949**
	NA- k_{d11}	0.01865	0.01869	0.02529	
	NA- k_{f11}	1.86500	1.86637	2.32216	0.989
	NA- k_{d12}	0.01865	0.01869	0.01877	
	NA- k_{f12}	1.86500	1.86605	1.87377	0.990
	NA- k_{d13}	0.01865	0.01868	0.01858	
	NA- k_{f13}	1.86500	1.86581	1.85975	0.990
	NA- k_{d14}	0.01865	0.01868	0.01707	
	NA- k_{f14}	1.86500	1.86564	1.77492	0.990
	NA- k_{d15}	0.01865	0.01868	0.01855	
	NA- k_{f15}	1.86500	1.86554	1.83255	0.990
**2**	**RA- k_{d2}**	**0.01865**	**0.01868**	**0.01859**	
	**RA- k_{f2}**	**0.34780**	**0.34788**	**0.34581**	**0.949**
	NA- k_{d21}	0.01865	0.01868	0.02010	
	NA- k_{f21}	1.86500	1.86514	1.92687	0.990
	NA- k_{d22}	0.01865	0.01868	0.01931	
	NA- k_{f22}	1.86500	1.86512	1.88922	0.990
	NA- k_{d23}	0.01865	0.01868	0.01865	
	NA- k_{f23}	1.86500	1.86511	1.84430	0.990
	NA- k_{d24}	0.01865	0.01868	0.01772	
	NA- k_{f24}	1.86500	1.86509	1.77584	0.990
	NA- k_{d25}	0.01865	0.01868	0.01897	
	NA- k_{f15}	1.86500	1.86506	1.90001	0.990
**3**	**RA- k_{d3}**	**0.01865**	**0.01868**	**0.01867**	
	**RA- k_{f3}**	**0.34780**	**0.34783**	**0.34715**	**0.949**

Table S2. DynaFit analysis of simulation of Figure S2c,d in which the NA-bands were pooled and analysed as a single peak, The expected effective $k_f \approx (5^*(1/1.98)^{-1} = 0.396$ min^{-1}. RA-band data shown in bold. The microscopic processivity was calculated from $k_f/(k_f + k_d)$ at each step.

Repeat number	Rate constant	Input value (min^{-1})	DynaFit estimate	Microscopic Processivity
	NA- $k_{d01\text{-}03}$	0.019	0.017	
	NA- $k_{f01\text{-}03}$	3 @ 1.98	0.872	0.981
**1**	**RA- k_{d1}**	**0.019**	**0.019**	
	**RA- k_{f1}**	**0.348**	**0.346**	**0.948**
	NA- $k_{d11\text{-}15}$	0.019	0.015	
	NA- $k_{f11\text{-}15}$	5 @ 1.98	0.326	0.955
**2**	**RA- k_{d2}**	**0.019**	**0.018**	
	**RA- k_{f2}**	**0.348**	**0.348**	**0.950**
	NA- $k_{d21\text{-}25}$	0.019	0.016	
	NA- $k_{f21\text{-}25}$	5 @ 1.98	0.346	0.955
**3**	**RA- k_{d3}**	**0.019**	**0.017**	
	**RA- k_{f3}**	**0.348**	**0.338**	**0.951**
	NA- $k_{d31\text{-}35}$	0.019	0.016	
	NA- $k_{f31\text{-}35}$	5 @ 1.98	0.346	0.955
**4**	**RA- k_{d4}**	**0.019**	**0.017**	
	**RA- k_{f4}**	**0.348**	**0.336**	**0.951**
	NA- $k_{d41\text{-}45}$	0.019	0.016	
	NA- $k_{f41\text{-}45}$	5 @ 1.98	0.342	0.955
**5**	**RA- k_{d5}**	**0.019**	**0.017**	
	**RA- k_{f5}**	**0.348**	**0.332**	**0.952**
	NA- $k_{d51\text{-}55}$	0.019	0.016	
	NA- $k_{f51\text{-}55}$	5 @ 1.98	0.338	0.956
**6**	**RA- k_{d6}**	**0.019**	**0.016**	
	**RA- k_{f6}**	**0.348**	**0.325**	**0.953**
	NA- $k_{d61\text{-}65}$	0.019	0.015	
	NA- $k_{f61\text{-}65}$	5 @ 1.98	0.329	0.958
**7**	**RA- k_{d7}**	**0.019**	**0.015**	
	**RA- k_{f7}**	**0.348**	**0.313**	**0.955**

Table S3. DynaFit analysis of simulation of Figure S2e,f in which the NA-bands were pooled with adjacent RA-bands or ignored. The microscopic processivity was calculated from $k_f/(k_f + k_d)$ at each step.

Rate constant	Input value (min^{-1})	DynaFit estimate (RA- plus NA-)	DynaFit estimate (RA- only))	Microscopic Processivity (RA- plus NA-)	Microscopic Processivity (RA- only))
**RA- k_{d1}**	0.019	0.022	0.015		
**RA- k_{f1}**	0.18	0.209	0.292	0.904	0.952
**RA- k_{d2}**	0.019	0.017	0.011		
**RA- k_{f2}**	0.18	0.167	0.247	0.907	0.955
**RA- k_{d3}**	0.019	0.015	0.011		
**RA- k_{f3}**	0.18	0.155	0.249	0.910	0.958
**RA- k_{d4}**	0.019	0.014	0.013		
**RA- k_{f4}**	0.18	0.151	0.288	0.912	0.957
**RA- k_{d5}**	0.019	0.014	0.019		
**RA- k_{f5}**	0.18	0.147	0.387	0.915	0.954
**RA- k_{d6}**	0.019	0.012	0.037		
**RA- k_{f6}**	0.18	0.141	0.682	0.920	0.949
**RA- k_{d7}**	0.019	0.010	0.129		
**RA- k_{f7}**	0.18	0.131	2.191	0.932	0.944

Example Software Scripts

Script for simulation of telomerase activity using Berkeley Madonna

Text in grey boxes is generated automatically on entry of model

```
1: primer+enz <--> A
        RXN1 = K1f*primer*enz - K1r*A
        K1f = 0.1
        K1r = 0.02
        INIT A = 0
        INIT enz = 5
        INIT primer = 50
        d/dt(A) = +RXN1-RXN2
        d/dt(enz) = -RXN1+RXN3+RXN5+RXN7+RXN9+RXN11-RX
        d/dt(primer) = -RXN1
```

```
2: A <--> B
        RXN2 = K2f*A - K2r*B
        K2f = 0.18
        K2r = 0
        INIT B = 0
        d/dt(B) = +RXN2-RXN3-RXN
```

```
3: B <--> Bx+enz
        RXN3 = K3f*B - K3r*Bx*en
        K3f = 0.02
        K3r = 0.1
        INIT Bx = 0
        d/dt(Bx) = +RXN3
```

```
4: B <--> C
        RXN4 = K4f*B - K4r*C
        K4f = 0.18
        K4r = 0
        INIT C = 0
        d/dt(C) = +RXN4-RXN5-RXN
```

```
5: C <--> Cx+enz
        RXN5 = K5f*C - K5r*Cx*en
        K5f = 0.02
        K5r = 0.1
        INIT Cx = 0
        d/dt(Cx) = +RXN5
```

```
6: C <--> D
        RXN6 = K6f*C - K6r*D
        K6f = 0.18
        K6r = 0
        INIT D = 0
        d/dt(D) = +RXN6-RXN7-RXN
```

```
7: D <--> Dx+enz
        RXN7 = K7f*D - K7r*Dx*en
        K7f = 0.02
        K7r = 0.1
        INIT Dx = 0
        d/dt(Dx) = +RXN7
```

```
8: D <--> E
        RXN8 = K8f*D - K8r*E
        K8f = 0.18
        K8r = 0
        INIT E = 0
        d/dt(E) = +RXN8-RXN9-RXN
```

```
9: E <--> Ex+enz
        RXN9 = K9f*E - K9r*Ex*en
        K9f = 0.02
        K9r = 0.1
        INIT Ex = 0
        d/dt(Ex) = +RXN9
```

```
10: E <--> F
        RXN10 = K10f*E - K10r*
        K10f = 0.18
        K10r = 0
        INIT F = 0
        d/dt(F) = +RXN10-RXN1
```

```
11: F <--> Fx+enz
        RXN11 = K11f*F - K11r*Fx*en
        K11f = 0.02
        K11r = 0.1
        INIT Fx = 0
        d/dt(Fx) = +RXN11
```

```
12: enz+X <--> chase
        RXN12 = K12f*enz*X - K12r*chas
        K12f = 0.1
        K12r = 0.02
        INIT chase = 0
        INIT X = 0
        d/dt(chase) = +RXN12
        d/dt(X) = -RXN12
```

METHOD RK4 ; 4th order Runge-Kutta

; Note Berkeley Madonna uses uppercase K for rate constants (see Table below). The model can be extended by adding more sequential steps. Concentrations are in nM , time in minutes and rate constants in minute-1

; Although rate constants are defined in the initial entry of the model, they are redefined below so that they can be readily updated individually or in groups. This is useful for changing the model between RA-steps only, and resolved RA- and NA-steps.

K1f=0.1 ; labeled primer binding
K1r=0.02

K2f=0.18 ; even number constants refer to product elongation (kf in main text)
K3f=0.02 ; odd numbered constants refer to product dissociation (kd in main text)
K4f=K2f
K5F=K3f
K6f=K2f
K7f=K3f
K8f=K2f
K9f=K3f
K10f=K2f
K11f=K3f

K3r=K1f ; assume products rebind with same rate constant as primer, but this can be modified

```
K5r=K3r
K7r=K3r
K9r=K3r
K11r=K3r


K12f=K1f            ; chase with unlabeled primer
K12r=K1r

; Calculate total product (Bt) from sum of bound (B) + free (Bx)

Bt=B+Bx
Ct=C+Cx
Dtot=D+Dx           ; note Dt is a reserved function name
Et=E+Ex
Ft=F+Fx             ; the last product modeled, Fx in this case, represents a sink of unresolved products

; The model can be extended by adding more steps

; Simulation of chase with unlabeled primer (X)

chasetime = 5
jump = 10000                                    ; chase with 10 uM unlabeled primer
d/dt(X)=pulse(jump,chasetime,1000)-RXN12        ; Add semicolon ; in front of this statement to disable chase step

STARTTIME = 0
STOPTIME=150            ; Timebase = minutes for telomerase modeling
DT = 0.0002             ; integration time
DTOUT = 1               ; plot time interval
```

Example script for analysis of telomerase activity using DynaFit

```
Example script for fitting multistep consecutive Telomerase reaction with chase at 5 min


In this example, products B, C, D etc. refer to RA-bands. The NA-bands are ignored, but the model can be extended to include them, in which case the initial estimates of rate constants should be changed. All products are assumed to have the same rebinding kinetics as the initial primer (association rate constant = k1).

Data are input as normalized absolute concs (nM) as a .csv file containing time, concentration.
;________________________________________________________________________

[task]

   data = progress
   task = fit
   ;confidence = monte-carlo    ; Remove semicolon to activate this step

[mechanism]
            Primer + Enz <==> A               : k1      k-1
            A ---> B                          : k2
            B <==> Bx + Enz                   : k3      k1
            B ---> C                          : k4
            C <==> Cx + Enz                   : k5      k1
            C ---> D                          : k6
            D <==> Dx + Enz                   : k7      k1
            D ---> E                          : k8
            E <==> Ex + Enz                   : k9      k1
            E ---> F                          : k10
            F <==> Fx + Enz                   : k11     k1

            Enz + X ---> chase                : k100



[constants]

; initial estimates of rate constants – those marked ? are adjusted to fit
            k1 = 0.1
            k-1 = 0.01
            k2 = 0.1?
            k3 = 0.01?
            k4 = 0.1?
            k5 = 0.01?
            k6 = 0.1?
            k7 = 0.01?
            k8 = 0.1?
            k9 = 0.01?
            k10 = 0.1?
            k11 = 0.02

            k100 = 0.1    ; Preincubation for 5 min. Define Time 0 as chase time

[concentrations]

            Primer = 50
            Enz=5?

[data]
; Note data are entered as csv files reporting time,concentration. For experimental data extracted from gels, the band intensities can be converted to concentrations or entered with arbitrary units, in which case the response factor must be adjusted.

   directory ./test/file_name
   file      B.csv
            incubate Primer = 50, Enz = 5, dilute =1, time =5
            concentration X =10000
             response B = 1   | response Bx = 1    | label B

   file      C.csv
```

```
            incubate Primer = 50, Enz = 5, dilute =1, time = 5
            concentration X =10000
             response C = 1    | response Cx = 1      | label C

   file      D.csv
            incubate Primer = 50, Enz = 5, dilute =1, time = 5
            concentration X =10000
             response D = 1    | response Dx = 1      | label D

   file      E.csv
            incubate Primer = 50, Enz = 5, dilute =1, time = 5
            concentration X =10000
             response E = 1    | response Ex = 1      | label E


[output]
   directory ./test/output

[end]
;_______________________________________________________________________________
;_______________________________________________________________________________
RESULTS:
```

For further details see http://www.biokin.com/

Rate constant definitions

Each program has a unique way of naming and numbering rate constants. This table summaraises equivalent rate constants with respect to the nomenclature in the main text.

Step	Berkeley Madonna	DynaFit	Main Text (Scheme 2)	Notes
Primer + enz ←→ A	K1f	k1	k_{a0}	
	K1r	k-1	k_{d0}	
A → B	K2f	k2	k_{f0}	
	K2r			Assumed = 0
B ←→ Bx + enz	K3f	k3	k_{d1}	
	K3r	k-3 or k1	k_{-d1}	= k_{a0} in model
B → C	K4f	k4	k_{f1}	
	K4r			Assumed = 0
C ←→ Cx + enz	K5f	k5	k_{d2}	
	K5r	k-5 or k1	k_{-d2}	= k_{a0} in model
C → D	K6f	k6	k_{f2}	
	K6r			Assumed = 0
